# In-Person vs. Virtual: A Comparative Study of Teaching Methods in Nutritional Medicine

**DOI:** 10.3390/nu18050821

**Published:** 2026-03-03

**Authors:** Benjamin Caspar Raphael Trutwin, Jantje Eilers, Hans Joachim Herrmann, Markus Friedrich Neurath, Matthias Kohl, Yurdagül Zopf, Leonie Cordelia Burgard

**Affiliations:** 1 Hector-Center for Nutrition, Exercise and Sports, Department of Medicine 1, Universitätsklinikum Erlangen, Friedrich-Alexander-Universität Erlangen-Nürnberg (FAU), 91054 Erlangen, Germany; 2 Department of Medicine 1, Universitätsklinikum Erlangen, Friedrich-Alexander-Universität Erlangen-Nürnberg (FAU), 91054 Erlangen, Germany; 3 Deutsches Zentrum Immuntherapie (DZI), Universitätsklinikum Erlangen, Friedrich-Alexander-Universität Erlangen-Nürnberg (FAU), 91054 Erlangen, Germany; 4 Faculty III: Health, Medical and Life Sciences, University Furtwangen, 78054 Villingen-Schwenningen, Germany

**Keywords:** online learning, nutritional medicine, medical education, curricular innovation, learning outcomes, student perceptions

## Abstract

**Background/Objectives:** Nutritional medicine remains underrepresented in medical education despite its relevance across specialties. Online learning offers a resource-efficient option to address this gap, yet evidence on the effectiveness and acceptability of online learning modules (OLMs) is limited. **Methods:** In this exploratory randomized controlled single post-test trial, medical students were assigned to either an OLM or an in-person lecture (IPL) on nutritional medicine (n = 91, no a priori sample size calculation performed). After course completion, students took a knowledge test and completed a questionnaire on their learning experience. Group differences were analyzed using permutation Welch *t*-tests, Wilcoxon–Mann–Whitney tests, or Fisher’s exact tests, depending on variable characteristics, with α = 0.05. **Results:** OLM students achieved significantly higher test scores than IPL students (mean difference: 2.4 points on a 0–40 scale), resulting in differences in grade classification (*p* < 0.05). OLM was further rated more favorably regarding content delivery, overall course evaluation, and exam preparation (all *p* < 0.05), while self-reported attention, concentration, and involvement did not differ between groups. Flexibility, time savings, and convenience were the most frequently reported advantages of OLM over IPL. **Conclusions:** This study suggests that OLM in nutritional medicine may be associated with higher test performance and more favorable student evaluations compared to IPL. These findings highlight the potential of online learning as a scalable, resource-efficient approach that may help address persistent gaps in nutritional medicine education. Building on this evidence, future work should examine how such modules can be optimally integrated into medical curricula to complement existing teaching structures.

## 1. Introduction

The COVID-19 pandemic accelerated digitalization in higher education and greatly expanded the possibilities for online teaching [1,2]. What initially evolved as an emergency response has, in the post-pandemic era, transformed into new opportunities for resource-efficient and flexible complements to traditional face-to-face teaching [3,4].

This potential is particularly relevant for under-served subjects, where online teaching can provide specialized instructors and resources that may be unavailable locally. One such area is nutritional medicine within the medical curriculum [5]. Nutrition-related diseases account for a substantial proportion of the global burden of disease: in 2017, an estimated 22% of all adult deaths were attributable to dietary risk factors [6]. Despite its relevance across virtually all medical specialties, nutritional medicine remains underrepresented in medical education. Dedicated chairs for this subject are scarce, structured teaching is often limited to elective courses, and students consistently report insufficient knowledge, skills, and confidence to counsel patients on diet-related issues [5,7,8,9]. Data mirror this gap: a 2014 survey among medical schools in Western Europe found only 68.8% of 32 responding institutions to require nutrition education as part of their curriculum, with an average of only 23.7 h dedicated to the subject [10]. Taken together, this underscores a clear need for curricular innovation [5,11,12].

Despite growing interest in digital teaching, evidence specifically addressing online learning in nutritional medicine within medical education remains limited. Findings from broader medical education research show a heterogeneous picture regarding learning outcomes and student perceptions. Two systematic reviews and meta-analyses concluded that online learning can be at least as effective as, and in some cases superior to, traditional face-to-face teaching in improving medical students’ knowledge and skills [13,14], with higher overall satisfaction with online formats [13]. However, evidence from non-medical higher education indicates that effectiveness is strongly influenced by contextual factors such as learner characteristics and learning environment [15].

Student perceptions are equally nuanced. Dumm et al. [7] examined an online lecture series in nutritional medicine and reported high acceptance among medical students in Germany but emphasized students’ desire for greater interactivity and practical application within digital formats. In a post-pandemic retrospective comparison of virtual and in-person case-based active-learning groups at a U.S. medical school, fewer than one in ten students favored an exclusively online format, with students considering in-person learning more conducive to engagement and collaborative learning [16]. Similarly, Zhang reported that in-person formats were perceived to enhance concentration and foster a positive learning environment with stronger instructor–student and peer–peer interaction [17]. These divergent findings likely reflect differences in study and course design, digital learning experience, and teaching cultures, limiting the generalizability of existing evidence. 

Online teaching formats may represent a cost-effective option to help address the educational gap in nutritional medicine by providing scalable learning opportunities [18]. However, their implementation requires evidence that such formats are equally effective and acceptable compared with conventional face-to-face teaching [19,20]. To date, such data remains inconclusive.

Against this background, the aim of this study was to compare an asynchronous video-based online learning module with a conventional in-person lecture in nutritional medicine (subsequently referred to as OLM and IPL, respectively) under authentic learning conditions. Specifically, we examined whether (a) students’ learning performance and (b) perceptions differed between the teaching formats.

## 2. Materials & Methods

### 2.1. Study Design and Setting

The study was conducted as a randomized controlled trial with a single post-test design in which medical students were assigned to either an OLM or IPL class. After completing the teaching format, participants were asked to take a knowledge test and fill out a questionnaire in which they evaluated their learning experience. The study was performed at the Hector-Center for Nutrition, Exercise, and Sports, Department of Medicine 1, Universitätsklinikum Erlangen, Friedrich-Alexander-Universität Erlangen-Nürnberg in Germany. Data collection took place in December 2024.

The Ethics Committee of the Friedrich-Alexander-Universität Erlangen-Nürnberg (FAU) was consulted prior to study initiation (telephone consultation, 17 October 2024). The committee indicated that formal ethical approval was not required according to institutional regulations, as the project did not involve the collection of identifiable or sensitive personal data. No reference number was assigned, as the project was classified as not requiring review. Written confirmation is provided upon request. To ensure adherence to health research reporting standards, the CONSORT-guideline was followed in manuscript writing [21]. During the preparation of this work, the authors used ChatGPT-5.1 (OpenAI, San Francisco, CA, USA; www.chatgpt.com) to assist with English language refinement, given the authors’ non-native speaker status. All generated content was subsequently reviewed, revised, and approved by the authors, who take full responsibility for all aspects of the publication.

### 2.2. Participants and Recruitment

**Figure 1:**
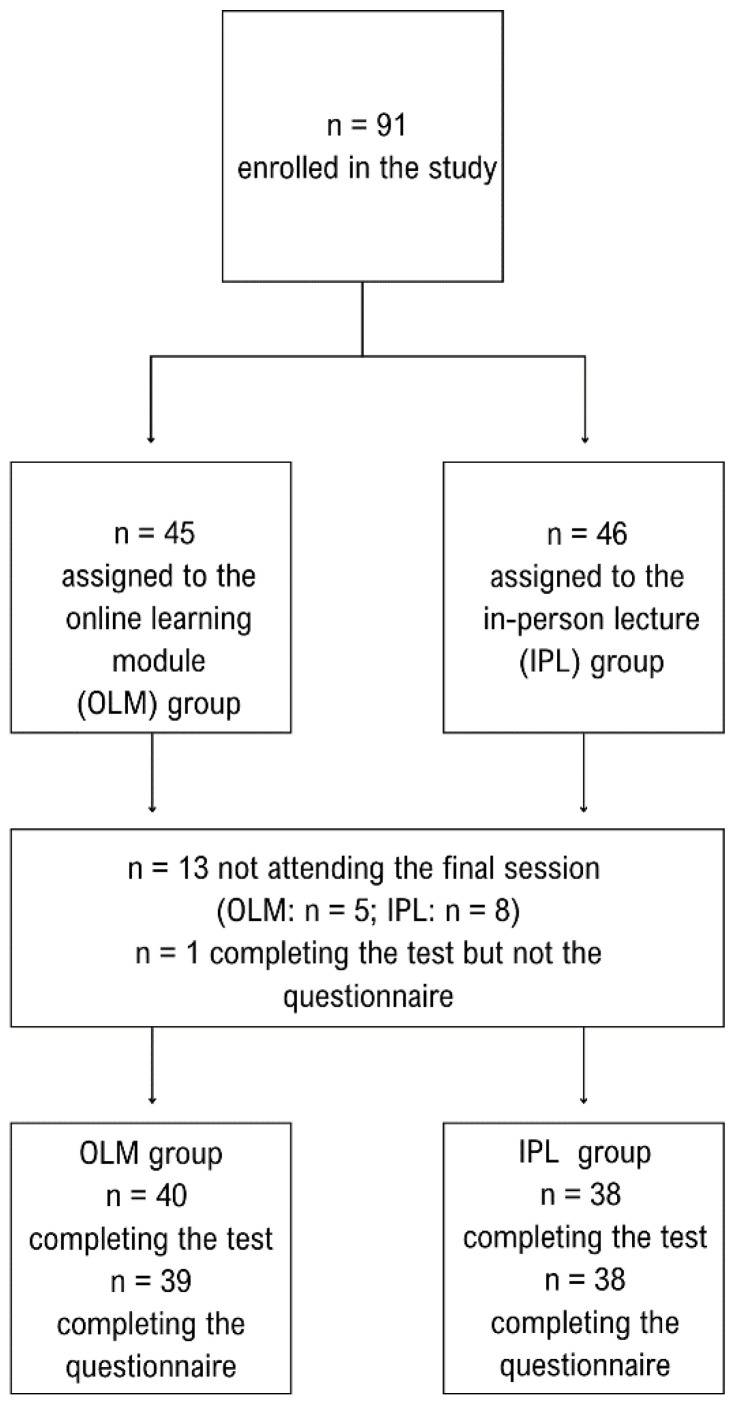
Participant flow chart.

A total of 91 medical students from the Friedrich-Alexander-Universität Erlangen-Nürnberg were included in the study. Eligibility criteria included German proficiency, access to an internet-enabled mobile device, and enrollment in the 6th–8th semester of medical school, which ensured a comparable academic background and sufficient prior knowledge in medicine to meaningfully participate in a nutritional medicine lecture. Recruitment was conducted via social media platforms and face-to-face recruitment at the medical faculty. The sample was randomized by drawing lots, performed by a member of the study team (the same personnel enrolled and assigned participants). Simple randomization without stratification or blocking was used. No allocation concealment was implemented. No blinding of participants, study personnel, or outcome assessors was conducted; however, the knowledge test consisted of single-choice questions with predefined correct answers, thereby limiting the potential for subjective bias. For questionnaire outcomes, design features such as requiring participants to specify numeric agreement values (0–100%) were used to standardize responses. Thirteen students (OLM: n = 5; IPL: n = 8) did not attend the assessment, and one OLM student completed the test but not the questionnaire. This resulted in 78 students completing the test and 77 completing both the test and questionnaire (see Figure 1). All study participants received an expense allowance of €5 in the form of a voucher. No a priori sample size calculation was performed as this was an exploratory pilot study.

### 2.3. Interventions

Both the OLM and the IPL group followed the same syllabus, covering key aspects of human metabolism (lipids, amino acids, carbohydrates) and nutritional interventions for selected diseases (e.g., colon cancer, diabetes mellitus). Students in the OLM group received the course content by means of an asynchronous video-based online module. This online module consisted of a series of 33 pre-recorded videos, each 3–10 min in length, totaling 176 min. The videos were produced by a medical student under close supervision of the project lead professor using the paper-slide technique, in which visual elements (figures, text, and illustrations) are manually animated on a flat surface. Narration was provided by two AI-generated voices (one female, one male). The videos were made available via a private YouTube link for two weeks (2–16 December 2024), after which access expired. Students could watch and rewatch the videos at their own pace. Students in the IPL group attended two 90 min in-person early afternoon lectures delivered by a Professor of Clinical and Experimental Nutritional Medicine at Friedrich-Alexander-Universität Erlangen-Nürnberg. The total lecture time equaled the duration of the online module. To maintain comparability, the lecture content was based on the OLM video scripts. An AI-based text generation tool [22] was used to adapt the video scripts into lecture scripts and a PowerPoint presentation. In order to match the traditional style of university lectures, metaphors and narrative elements were largely omitted, without changing or cutting any factual content. The lectures took place in the afternoon on Monday, 2 December 2024, and Wednesday, 11 December 2024. The sessions followed the standard format of medical lectures at the university. Each lecture included interactive elements, such as questions directed at the students, and concluded with time for discussion. Students were encouraged to ask questions throughout the lecture and to take notes. Participants’ adherence (online and in-person) was not recorded, and hence no minimum attendance was required to participate in the final assessment.

### 2.4. Outcome Measures

A supervised test was administered to evaluate learning outcomes accompanied by a questionnaire to assess learning experience. The test took place on the 16th of December 2024 and consisted of 40 single-choice questions of varying difficulty to be completed within 60 min. The study participants received one point for each correctly answered question. Both the structure and grading of the test mirrored those of a standard internal medicine examination at the Friedrich-Alexander-Universität Erlangen-Nürnberg. The derived test score is a continuous variable ranging from 1 to 40 points. Raw test scores were subsequently converted into grades. Grades were assigned on a scale from 1.0 (best) to 6.0 (worst) based on four-point intervals; for example, 36–40 points corresponded to a grade of 1.0 (American letter grade A). Following the test, participants completed a questionnaire designed to evaluate their learning experience. The questionnaire comprised two sections: (1) demographics, prior knowledge, and interest in nutritional medicine and (2) evaluation of the respective teaching format, including perceived advantages, concentration, motivation, and overall satisfaction. Online participants were additionally asked about video usage and learning behavior. Items included a mix of dichotomous (yes/no), multiple-choice, and agreement-based questions. The questionnaire was developed for internal evaluation purposes. Detailed documentation regarding its development and formal validation is not available. Note that neither the lectures (online or in-person) nor the tests were part of the regular medical curriculum but were designed in a mock format specifically for this study.

### 2.5. Statistical Analysis

Statistical analyses were performed using R (version 4.4.3, R Foundation for Statistical Computing, Vienna, Austria; www.r-project.org/). Descriptive statistics are reported as frequency, percentage, arithmetic mean, standard deviation, median, and interquartile range, as appropriate. Potential group differences in test outcomes were analyzed using the permutation Welch *t*-test and/or the Wilcoxon–Mann–Whitney test [23], depending on variable distribution. For the questionnaire data, either the Wilcoxon–Mann–Whitney test (continuous variables) or Fisher’s exact test was used (categorical variables). For the test outcomes, effect sizes (Cohen’s d for parametric tests and r for non-parametric tests) and corresponding 95% confidence intervals for group differences were reported in addition to *p*-values. A two-sided significance level of α = 0.05 was set for all tests. Details on missing data for all variables are provided in Supplementary Table S1. Missing values were not imputed; analyses were conducted using available-case data, resulting in varying sample sizes across individual tests.

## 3. Results

The sample characteristics are displayed in Table 1. The students included in the study were between 20 and 29 years of age. While students assigned to the OLM group were in a significantly higher semester of study as compared to their IPL peers, demographic characteristics were otherwise comparable between groups.

**Figure 2:**
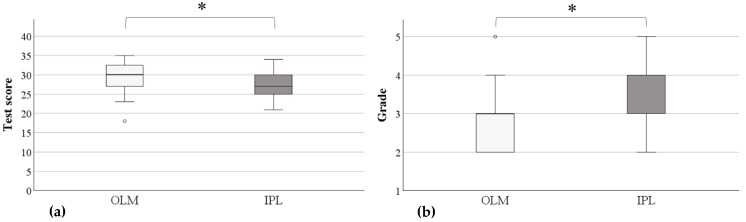
Boxplots of test scores (**a**) and grades (**b**) for the online learning module (OLM); n = 40) and the in-person lecture (IPL; n = 38). Boxplots show medians and interquartile ranges; whiskers represent 1.5 × IQR. Significant Wilcoxon–Mann–Whitney test results (*p* < 0.05) are marked with an asterisk. Note: Performance is better the higher the test score and the lower the grade. Details on missing data for all variables are provided in Supplementary Table S1.

The median test score was 30 points in the OLM group and 27 points in the IPL group (see Figure 2). Both the permutation Welch *t*-test and the Wilcoxon–Mann–Whitney test indicated a significant difference in test performance between groups, with students in the OLM group achieving higher scores. The mean difference was 2.4 points (95% CI [0.7, 4.0]; t = −2.89, *p* = 0.004), corresponding to a medium effect size (Cohen’s d = 0.65). The Wilcoxon–Mann–Whitney test confirmed this result (difference in location = 2.5 points, 95% CI [0.0, 5.0]; W = 464, *p* = 0.003, effect size r = 0.34). Although the median grade was 3.0 in both groups, the Wilcoxon–Mann–Whitney test indicated significantly higher grades in the OLM group (difference in location = 0.5, 95% CI [0.0, 1.0]; W = 998, *p* = 0.011; effect size r = 0.29).

**Figure 3:**
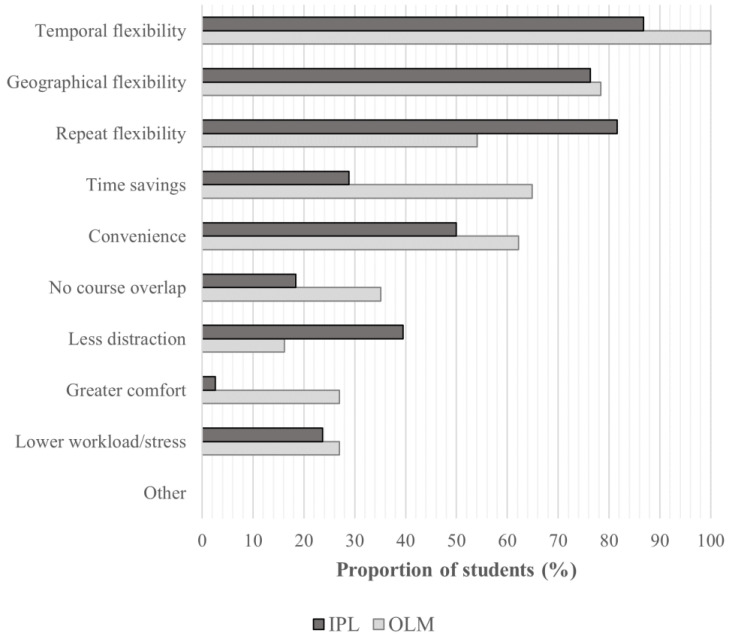
Advantages of online learning over traditional lectures reported by students in the online learning module (OLM; n = 37, 2 missing responses) and the in-person lecture (IPL; n = 38).

Findings regarding student perceptions are summarized in Table 2, with inferential statistics provided in Supplementary Table S2. The perceived suitability of the course format for conveying the topics was higher in the OLM group compared with the IPL group. Students also provided higher overall ratings of the course format and the course itself when taught via the online module. A pronounced difference in means was observed for the adequacy of exam preparation, with higher ratings in the OLM group compared to the IPL group. Consistent with this, a greater proportion of students in the OLM group indicated openness to taking the same format again for exam preparation. No differences were found for self-perceived levels of attentiveness, concentration, or involvement. The most frequently reported advantages of online learning in both groups were flexibility (particularly temporal and geographical flexibility), time savings, and convenience, while the most frequently reported disadvantages were social isolation and reduced discussion/interaction. The reported advantages of online learning over traditional lectures are further illustrated in Figure 3.

Further evaluations within the OLM group are summarized in Table 3. Students’ evaluations of the video quality and format were consistently positive. The visualization and presentation quality received ratings around 80% on a scale from 0% (“very poor”) to 100% (“very good”). Around three in four students used the pause function, typically only for brief periods, and more than half revisited selected parts of the videos using the repeat function. Self-paced learning was perceived as beneficial for retention and motivation (89.7% and 82.1%, respectively). In addition, many students considered the OLM format to allow more effective use of study time (82.1%) and to support better attention (74.4%). Students spent on average 3.46 h studying, exceeding the total lecture time scheduled for the IPL group. Videos were primarily watched at home and across a broad range of daytime and early evening hours. Nearly nine in ten students (87.2%) expressed willingness to use the OLM format in other subjects.

## 4. Discussion

Our findings suggest that online learning may offer a viable alternative to traditional face-to-face lectures in nutritional medicine, with higher test scores in the OLM group, reflected in grade classifications. Beyond objective performance, students also rated the OLM more favorably across key aspects of teaching quality, particularly content delivery, overall course evaluation, and exam preparation. This was observed despite similar self-reported levels of attention, concentration, and involvement between groups. Students particularly valued the OLM for its flexibility, convenience, and time savings.

Drawing on established educational theories, several pedagogical aspects may explain the higher test performance observed in the OLM group. Digital learning environments provide autonomy and convenience (i.e., by enabling self-paced learning and the intentional structuring of breaks), which increase learning effectiveness and retention through enhanced cognitive and emotional engagement [24,25]. The flexibility of asynchronous online formats further allows students to engage with course material during periods of peak alertness, better aligning study activities with individual chronotypes [26]. Additionally, OLMs enable repeated exposure to learning content, whereas in-person teaching typically provides a one-time learning experience. Students in the OLM group reported a longer total study time compared with the scheduled lecture duration in the IPL group (approximately 30 min, corresponding to ~15%), likely reflecting the ability to access and replay the video content flexibly. Moreover, online learning reduces extrinsic stressors, including commuting time, crowded lecture halls, and the balancing of academic, personal, and professional commitments. By alleviating these factors, online formats may free up cognitive and emotional resources, allowing students to devote more capacity to the learning process itself, consistent with principles of cognitive load theory [27].

Regarding student perceptions, OLM students reported valuing the format for its flexibility, convenience, and attributed time savings. Ahmed et al. [24] highlighted these aspects as key drivers for student motivation in e-learning environments. This is consistent with findings from fully online higher education, where course designs that support students’ time management and self-regulated pacing are associated with greater satisfaction and higher academic achievement [28]. Although OLM and IPL students reported similar levels of self-perceived attentiveness, three-quarters of OLM students reported feeling more attentive in the OLM compared with traditional lectures. This discrepancy may reflect the greater autonomy and flexibility in OLM, which could create a positive perception bias, with students feeling more engaged due to control over their learning process. Of note, the observed advantages of OLM, both in test performance and student perceptions, may be related to specific design characteristics rather than to the online format per se. In particular, the enhanced sense of autonomy observed in the OLM group may primarily reflect the asynchronous nature of the format rather than online delivery as such. Supporting this interpretation, temporal flexibility was the most frequently reported advantage of online learning over traditional lecture formats across both groups. Likewise, differences in social interaction may be more strongly influenced by synchronicity than by delivery mode. Future research should therefore explicitly disentangle the effects of delivery mode (online vs. in-person) from those of synchronicity (synchronous vs. asynchronous). Importantly, the questionnaire data should be interpreted as descriptive and hypothesis-generating in light of the study’s exploratory nature, multiple comparisons, missing data, and the use of a non-validated questionnaire.

Overall, most of the discussed pedagogical and student-reported advantages of OLM are directly or indirectly linked to an enhanced learner’s sense of autonomy. This suggests that the greater autonomy enabled by online learning may underlie the observed differences between OLM and IPL. This interpretation aligns with Self-Determination Theory, which identifies autonomy—defined as *“a sense of initiative and ownership in one’s actions”*—alongside competence and relatedness, as central to students’ motivation, engagement, and well-being [29].

This study’s findings should not be interpreted in terms of an either-or decision between online and in-person teaching. Rather, the central question is how gaps in the curriculum can be addressed most efficiently with the resources available. In this sense, the issue is not online versus face-to-face instruction, but how both modalities can be combined to provide high-quality, resource-efficient education. The call for an integrative approach between online and offline teaching aligns with broader evidence in medical education, which shows that blended learning can outperform both purely in-person and fully online formats. Zhang and colleagues compared the effectiveness of blended, online, and offline learning modes among 2100 undergraduate medical students in China. The blended group achieved higher exam scores and pass rates than both the in-person and online groups, with 71.6% of participants preferring this method [17]. Similarly, Malta et al. [16] found that a hybrid model was preferred over fully virtual or in-person active learning groups in a US medical school. These findings suggest that online and offline teaching should be viewed as complementary tools that, when strategically combined, may offer the most effective learning environment.

In this context, it should be further acknowledged that the effectiveness of online teaching may strongly depend on the type of educational content. While primarily knowledge-based topics—such as metabolic pathways and nutritional guidelines—can be efficiently conveyed through OLM, other competencies, including clinical decision-making and patient communication skills, may benefit far more from in-person classes. In the present study, the absence of disadvantages in attention or involvement ratings for OLM suggests that such factors were not critical for the learning objectives addressed. Yet, their relevance should be considered when generalizing these findings to other areas of medical education.

Looking beyond the comparison of teaching formats, the broader structural context of nutritional medicine education must be acknowledged. Educational resources in this field remain limited, and specialized faculty are scarce. In Germany, to the best of the authors’ knowledge, only five of the forty medical faculties have a full professorship explicitly dedicated to nutritional medicine. Moreover, a nationwide survey of 1500 medical students showed that 88% estimated receiving fewer than 12 h of teaching in nutritional medicine or related topics throughout their studies [7]. Similar deficits have been described across Europe, prompting initiatives such as the ESPEN Nutrition Education in Medical Schools (NEMS) project, which aims to develop a harmonized core curriculum and promote resource sharing [30]. Against this backdrop, the question at this point in time is not whether digital methods should be used, but how they can be leveraged most effectively. With so few experts available, digital learning is essential to multiply limited expertise and to make high-quality teaching accessible to students beyond a single institution. Furthermore, online courses have the potential to extend beyond medical education and national borders by enabling the international dissemination of expertise and strengthening interdisciplinary training pathways. In this context, networked university structures—such as the European University Alliances—provide an institutional framework that enables cross-institutional participation in online courses and facilitates the mutual recognition and transfer of academic credits across participating higher education institutions [31].

This study has several limitations. First, voluntary enrollment may have resulted in a cohort with overall higher motivation than typically observed in routine curricular settings, which could limit ecological validity. Second, the study was conducted at a single German university with a relatively small sample size, which restricts generalizability and causal inference. Third, the post-test-only design limits causal attribution of observed differences to the teaching format. In addition, students in the OLM group had a higher mean number of semesters completed compared to their IPL peers. As test results and questionnaire data were collected anonymously on separate forms, individual-level adjustment for semester was not feasible. Although nutritional medicine is not systematically covered in the medical curriculum, residual confounding by academic seniority cannot be excluded. Accordingly, the findings should be interpreted with appropriate caution. In addition, a substantial proportion of the findings are based on self-reports, which are susceptible to response or novelty bias and may be influenced by pre-existing preferences for online learning. Moreover, the questionnaire used for evaluation was originally developed for internal purposes and was not formally validated. Additionally, missing data points and multiple comparisons limit the robustness of findings. Consequently—while providing useful insights—the questionnaire results should be cautiously interpreted and not given equal weight as objective learning outcomes. Furthermore, the OLM used in this study was developed and recorded by a medical student without prior expertise in didactics or video production; however, it was prepared in close collaboration with and under the supervision of the project’s lead professor. While this may limit generalizability, it also indicates that the potential of online learning may be even greater when supported by didactic guidance and higher-quality production resources. Finally, educational outcomes and learner perceptions are highly context-dependent, and controlling for format-specific characteristics is an inherent challenge in educational research. Aspects such as the use of AI-generated voices or the familiarity of the YouTube hosting environment represent only some of the specific contextual factors that may have shaped the results. These limitations should be considered when interpreting the present findings and their applicability to other educational contexts.

## 5. Conclusions

This exploratory study suggests that an asynchronous online learning format in nutritional medicine may be associated with higher test performance and more favorable student evaluations compared to traditional face-to-face lectures. Given the limited teaching capacity in this field, these findings highlight online learning as a scalable and resource-efficient approach to addressing persistent gaps in underserved curricular areas. Building on existing evidence, future work should explore how online learning in nutritional medicine can be optimally integrated into medical curricula and embedded within emerging cross-institutional online education frameworks. Overall, the findings emphasize that online learning should be viewed as a complementary tool rather than a workaround. When strategically combined with offline learning, it may provide the most effective learning environment in terms of both formal learning outcomes and student satisfaction.

## Figures and Tables

**Table 1:** Sample characteristics (based on questionnaire data, n = 77).

	OLM (n = 39)	IPL (n = 38)	*p*-Value
Age (years), mean ± SD	23.39 ± 1.96	23.53 ± 1.44	0.386
Sex, n (%)			
Male	20 (55.6)	15 (45.5)	0.473
Female	16 (44.4)	18 (54.5)	
Semester of study ^1^, mean ± SD	7.69 ± 0.58	7.27 ± 0.80	0.016
Prior knowledge of nutritional medicine ^2^, mean ± SD	0.59 ± 0.19	0.56 ± 0.20	0.330
Interest in nutritional medicine ^2^, mean ± SD	0.69 ± 0.20	0.74 ± 0.16	0.276
Preference for online learning over traditional lectures, n (%)	29 (76.3)	26 (68.4)	0.609
Learning type ^3^, n (%)			0.335
Visual learner	25 (41.7)	16 (26.6)	
Reading/writing learner	16 (26.7)	18 (30.0)	
Auditory learner	4 (6.7)	3 (5.0)	
Combination of several learning types	13 (21.7)	20 (33.3)	
Other	1 (1.7)	3 (5.0)	

One participant did not complete the questionnaire and was therefore not included in the corresponding analyses. OLM = online learning module; IPL = in-person lecture; SD = standard deviation. Significant *p*-values (α = 0.05) are displayed in bold. For categorical variables, Fisher’s exact test was applied; for continuous variables, the Wilcoxon–Mann–Whitney test was used. Details on missing data for all variables are provided in Supplementary Table S1. ^1^ Out of the 12 semesters of standard study duration in Germany. ^2^ Scale from 0% (“very poor”) to 100% (“very good”). ^3^ Multiple-choice questions.

**Table 2:** Students’ perceptions of the teaching formats, descriptive statistics.

	OLM (n = 39)	IPL (n = 38)
Suitability of the course format for conveying the topics ^1^, mean ± SD	0.77 ± 0.16	0.65 ± 0.19
Overall rating of the course format ^1^, mean ± SD	0.74 ± 0.21	0.65 ± 0.18
Overall rating of the course ^1^, mean ± SD	0.79 ± 0.16	0.70 ± 0.18
Adequacy of exam preparation ^1^, mean ± SD	0.79 ± 0.17	0.45 ± 0.21
Openness to taking the course format again for exam preparation, n (%)	32 (82.1)	18 (48.6)
Perceived advantages of online learning over traditional lectures ^2^, n (%)		
Temporal flexibility	37 (100)	33 (86.8)
Geographical flexibility	29 (78.4)	29 (76.3)
Repeat flexibility	20 (54.1)	31 (81.6)
Time savings	24 (64.9)	11 (28.9)
Convenience	23 (62.2)	19 (50.0)
No course overlap	13 (35.1)	7 (18.4)
Less distraction	6 (16.2)	15 (39.5)
Greater comfort	10 (27.0)	1 (2.6)
Lower workload/stress	10 (27.0)	9 (23.7)
Other	0 (0.0)	0 (0.0)
Perceived disadvantages of online learning over traditional lectures ^2^, n (%)		
Social isolation	24 (61.5)	36 (94.7)
Less discussion/interaction	24 (61.5)	29 (76.3)
Questions on the topic remain unanswered	12 (30.8)	23 (60.5)
Financial or technical access barriers	3 (7.7)	8 (21.1)
Greater distraction	19 (48.7)	14 (36.8)
Other	2 (5.1)	0 (0.0)
Self-perceived level of attentiveness during the course ^1^, mean ± SD	0.62 ± 0.22	0.60 ± 0.19
Self-perceived level of concentration during the course ^1^, mean ± SD	0.61 ± 0.21	0.55 ± 0.17
Self-perceived level of involvement during the course ^1^, mean ± SD	0.45 ± 0.29	0.57 ± 0.26

OLM = online learning module; IPL = in-person lecture; SD = standard deviation. Details on missing data for all variables are provided in Supplementary Table S1. ^1^ Scale from 0% (“very poor”) to 100% (“very good”). ^2^ Multiple choice question.

**Table 3:** Students’ experiences and perceptions in the OLM group (n = 39).

**Video quality and format**	
Quality of video visualization and presentation ^1^, mean ± SD	0.78 ± 0.18
Appropriateness of video length ^1^, mean ± SD	0.82 ± 0.20
Preferred video duration (min), mean ± SD	8.7 ± 6.0
**Learning behavior and use of flexible video functions**	
Use of pause function, n (%)	30 (76.9)
Average frequency of pauses if taken, mean ± SD	2.0 ± 2.1
Use of repeat function, n (%)	22 (56.4)
**Perceived learning effectiveness and motivation**	
Improved retention at self-paced viewing, n (%)	35 (89.7)
More effective time use compared to traditional lecture, n (%)	32 (82.1)
Increased motivation through self-paced viewing, n (%)	32 (82.1)
Better attention compared to traditional lecture, n (%)	29 (74.4)
**Study behavior**	
Total study time (hours), mean ± SD	3.46 ± 2.36
Typical time of day for video use ^2^, n (%)	
6 a.m.–11 a.m.	9 (17.3)
11 a.m.–1 p.m.	12 (23.1)
1 p.m.–3 p.m.	10 (19.2)
3 p.m.–6 p.m.	8 (15.4)
6 p.m.–10 p.m.	11 (21.2)
10 p.m.–6 a.m.	2 (3.8)
Location of video use ^2^, n (%)	
At home	33 (73.3)
University facilities (lecture halls, libraries, etc.)	8 (17.8)
Public places (park, café, etc.)	4 (8.9)
**Preference for using OLM in other subjects, n (%)**	34 (87.2)

SD = standard deviation; OLM = online learning module. Details on missing data for all variables are provided in Supplementary Table S1. ^1^ Scale from 0% (“very poor”) to 100% (“very good”). ^2^ Multiple-choice question.

## Data Availability

The data presented in this study are available on request from the corresponding author. (The data are not publicly available due to institutional restrictions.)

## Supplementary Materials

The following supporting information can be downloaded at: https://www.mdpi.com/article/10.3390/nu18050821/s1, Table S1. Overview of missing responses in the questionnaire data; Table S2. Students’ perceptions of the teaching formats with corresponding inferential statistics comparing the OLM (n = 39) and IPL (n = 38) groups; Table S3: CONSORT checklist [21].
