# Supplementary material for: In-Person vs. Virtual: A Comparative Study of Teaching Methods in Nutritional Medicine
Source: Nutrients. 2026 Mar 3;18(5):821. doi: 10.3390/nu18050821 (PMC12986958; doi:10.3390/nu18050821)
Supplement: Supplementary file 1 [file nutrients-18-00821-s001.zip › nutrients-4117014-supplementary.pdf]

Supplementary Material

Table S1: Overview of missing responses in the questionnaire data

n	OLM	IPL
Age (years)	3	6
Sex	3	5
Semester of study	3	5
Prior knowledge of nutritional medicine	1	3
Interest in nutritional medicine	1	2
Preference for online learning over traditional lectures	1	0
Suitability of the course format for conveying topics	1	2
Overall rating of the course format	1	1
Overall rating of the videos	1	2
Adequacy of exam preparation	3	5
Openness to taking the course format again for exam preparation	0	1
Perceived advantages of online learning over traditional lectures	2	0
Self-perceived level of attentiveness during the course	0	1
Self-perceived level of concentration during the course	1	1
Self-perceived level of involvement during the course	1	2

OLM = online learning module; IPL = in-person lecture

Table S2. Students' perceptions of the teaching formats with corresponding inferential statistics comparing the OLM (n = 39) and IPL (n = 38) groups.

**Questionnaire item**	***p*-value**
Suitability of the course format for conveying the topics[1], mean ± SD	0.003
Overall rating of the course format[1], mean ± SD	0.007
Overall rating of the course[1], mean ± SD	0.017
Adequacy of exam preparation[1], mean ± SD	< 0.001
Openness to taking the course format again for exam preparation, n (%)	0.002
Perceived advantages of online learning over traditional lectures[2], n (%)	0.009
Temporal flexibility	
Geographical flexibility	
Repeat flexibility	
Time savings	
Convenience	
No course overlap	
Less distraction	
Greater comfort	
Lower workload/stress	
Other	
Perceived disadvantages of online learning over traditional lectures[2], n (%)	0.168
Social isolation	
Less discussion/interaction	
Questions on the topic remain unanswered	
Financial or technical access barriers	
Greater distraction	
Other	
Self-perceived level of attentiveness during the course[1], mean ± SD	0.501
Self-perceived level of concentration during the course[1], mean ± SD	0.106
Self-perceived level of involvement during the course[1], mean ± SD	0.071

OLM = online learning module; IPL = in-person lecture; SD = standard deviation.
Details on missing data for all variables are provided in Supplementary **Table S1**.
For categorical variables, Fisher's exact test was applied; for continuous variables, the Wilcoxon–Mann–Whitney test was used.
[1] Scale from 0 % ("very poor") to 100 % ("very good").
[2] Multiple choice question.

Table S3: CONSORT checklist [1]

Section/topic	No	CONSORT 2025 checklist item description	Reported on page no.
**Title and abstract**			
Title and structured abstract	1a	Identification as a randomised trial	1&3
	1b	Structured summary of the trial design, methods, results, and conclusions	1
**Open science**			
Trial registration	2	Name of trial registry, identifying number (with URL) and date of registration	Not registered.
Protocol and statistical analysis plan	3	Where the trial protocol and statistical analysis plan can be accessed	Upon request from the corres-ponding author.
Data sharing	4	Where and how the individual de-identified participant data (including data dictionary), statistical code and any other materials can be accessed	Upon request from the corres-ponding author.
Funding and conflicts of interest	5a	Sources of funding and other support (eg, supply of drugs), and role of funders in the design, conduct, analysis and reporting of the trial	12
	5b	Financial and other conflicts of interest of the manuscript authors	12
**Introduction**			
Background and rationale	6	Scientific background and rationale	2
Objectives	7	Specific objectives related to benefits and harms	2
**Methods**			
Patient and public involvement	8	Details of patient or public involvement in the design, conduct and reporting of the trial	Not applicable.
Trial design	9	Description of trial design including type of trial (eg, parallel group, crossover), allocation ratio, and framework (eg, superiority, equivalence, non-inferiority, exploratory)	3
Changes to trial protocol	10	Important changes to the trial after it commenced including any outcomes or analyses that were not prespecified, with reason	None.
Trial setting	11	Settings (eg, community, hospital) and locations (eg, countries, sites) where the trial was conducted	3&4
Eligibility criteria	12a	Eligibility criteria for participants	3
	12b	If applicable, eligibility criteria for sites and for individuals delivering the interventions (eg, surgeons, physiotherapists)	Not applicable.
Intervention and comparator	13	Intervention and comparator with sufficient details to allow replication. If relevant, where additional materials describing the intervention and comparator (eg, intervention manual) can be accessed	3–5

Section/topic	No	CONSORT 2025 checklist item description	Reported on page no.
Outcomes	14	Prespecified primary and secondary outcomes, including the specific measurement variable (eg, systolic blood pressure), analysis metric (eg, change from baseline, final value, time to event), method of aggregation (eg, median, proportion), and time point for each outcome	5
Harms	15	How harms were defined and assessed (eg, systematically, non-systematically)	The study does not entail any specific risks to participants.
Sample size	16a	How sample size was determined, including all assumptions supporting the sample size calculation	Given the exploratory nature of the study, no formal sample size calculation was performed.
	16b	Explanation of any interim analyses and stopping guidelines	None performed.
Randomisation:			
Sequence generation	17a	Who generated the random allocation sequence and the method used	3
	17b	Type of randomisation and details of any restriction (eg, stratification, blocking and block size)	3
			**Reported on page no.**
Allocation concealment mechanism	18	Mechanism used to implement the random allocation sequence (eg, central computer/telephone; sequentially numbered, opaque, sealed containers), describing any steps to conceal the sequence until interventions were assigned	No allocation concealment performed.
Implementation	19	Whether the personnel who enrolled and those who assigned participants to the interventions had access to the random allocation sequence	3
Blinding	20a	Who was blinded after assignment to interventions (eg, participants, care providers, outcome assessors, data analysts)	No blinding conducted.
	20b	If blinded, how blinding was achieved and description of the similarity of interventions	No blinding conducted.
Statistical methods	21a	Statistical methods used to compare groups for primary and secondary outcomes, including harms	5
	21b	Definition of who is included in each analysis (eg, all randomised participants), and in which group	3&4
	21c	How missing data were handled in the analysis	5

Section/topic	No	CONSORT 2025 checklist item description	Reported on page no.
	21d	Methods for any additional analyses (eg, subgroup and sensitivity analyses), distinguishing prespecified from post hoc	No additional analyses performed.
**Results**			
Participant flow, including flow diagram	22a	For each group, the numbers of participants who were randomly assigned, received intended intervention, and were analysed for the primary outcome	3&4
	22b	For each group, losses and exclusions after randomisation, together with reasons	3&4
Recruitment	23a	Dates defining the periods of recruitment and follow-up for outcomes of benefits and harms	4&5
	23b	If relevant, why the trial ended or was stopped	Not applicable.
Intervention and comparator delivery	24a	Intervention and comparator as they were actually administered (eg, where appropriate, who delivered the intervention/comparator, how participants adhered, whether they were delivered as intended (fidelity))	4&5
	24b	Concomitant care received during the trial for each group	Not applicable.
Baseline data	25	A table showing baseline demographic and clinical characteristics for each group	5&6
Numbers analysed, outcomes and estimation	26	For each primary and secondary outcome, by group:	
		● the number of participants included in the analysis	3&4
		● the number of participants with available data at the outcome time point	3&4
		● result for each group, and the estimated effect size and its precision (such as 95% confidence interval)	5–9
		● for binary outcomes, presentation of both absolute and relative effect size	Not applicable.
Harms	27	All harms or unintended events in each group	Not applicable.
Ancillary analyses	28	Any other analyses performed, including subgroup and sensitivity analyses, distinguishing pre-specified from post hoc	Not applicable.
**Discussion**			
Interpretation	29	Interpretation consistent with results, balancing benefits and harms, and considering other relevant evidence	9–12
Limitations	30	Trial limitations, addressing sources of potential bias, imprecision, generalisability, and, if relevant, multiplicity of analyses	11

[1] Hopewell, S.; Chan, A.W.; Collins, G.S.; Hróbjartsson, A.; Moher, D.; Schulz, K.F.; Tunn, R.; Aggarwal, R.; Berkwits, M.; Berlin, J.A., et al. CONSORT 2025 statement: updated guideline for reporting randomised trials. *BMJ* **2025**, *389*, e081123, doi:10.1136/bmj-2024-081123.
